# An unexpected case of hypothyroidism in a young female patient undergoing first-line anti-tuberculosis therapy for pulmonary tuberculosis

**DOI:** 10.1093/omcr/omaf008

**Published:** 2025-03-28

**Authors:** Haroon Ahmed Khan, Uooja Devi, Muhammad Faheem Iqbal, Ali Aamir

**Affiliations:** Department of General Surgery, Institution: Dr Ruth KM Pfau Civil Hospital, Mission Road, New Labour Colony, Nanakwara, Karachi 74200, Pakistan; Department of General Surgery, Institution: Dr Ruth KM Pfau Civil Hospital, Mission Road, New Labour Colony, Nanakwara, Karachi 74200, Pakistan; Department of Internal Medicine, Institution: Dr Ruth KM Pfau Civil Hospital, Mission Road, New Labour Colony, Nanakwara, Karachi 74200, Pakistan; Department of General Surgery, Institution: Dr Ruth KM Pfau Civil Hospital, Mission Road, New Labour Colony, Nanakwara, Karachi 74200, Pakistan

**Keywords:** Critical care medicine, Endocrinology and metabolism, Gastroenterology, General Surgery, Infectious diseases and tropical medicine, Respiratory Disorders

## Abstract

Hypothyroidism, once considered a rare adverse effect of anti-tuberculosis (TB) medication, is increasingly observed in patients undergoing second-line therapy for multidrug-resistant tuberculosis (MDR-TB). This study discusses a case involving a 17-year-old female patient who presented to the emergency department with sub-acute intestinal obstruction secondary to pulmonary TB. She underwent an exploratory laparotomy, during which routine investigations revealed elevated thyroid-stimulating hormone (TSH) levels. A week later, a second laparotomy was performed under general anaesthesia. Postoperatively, facial puffiness was noted, prompting a repeat TSH test, which indicated a significant increase from the initial levels. The diagnosis was revised to hypothyroidism potentially related to anti-tuberculosis therapy (ATT). While existing literature predominantly associates hypothyroidism with second-line anti-TB medications in MDR-TB patients, this study suggests a potential link between first-line ATT and hypothyroidism. This finding underscores the importance of monitoring TSH levels in patients on first-line anti-TB drugs.

## Introduction

Anti-tuberculosis (TB) medications frequently induce hypothyroidism, particularly in individuals with multidrug-resistant tuberculosis (MDR-TB). Research from Karnataka, India indicated that approximately 23% of MDR-TB patients with accurate thyroid-stimulating hormone (TSH) readings develop hypothyroidism during anti-TB treatment [1]. Other studies have reported a 17% incidence of hypothyroidism induced by antitubercular drugs, typically manifesting two–three months into treatment and resolving four–six weeks after cessation (2). This condition is believed to result from the suppression of thyroperoxidase activity, which inhibits thyroid hormone synthesis [2].

Rifampin, ethionamide, prothionamide, and para-aminosalicylic acid are among the anti-TB medications most commonly associated with hypothyroidism [3, 4]. Although there is substantial evidence linking second-line anti-TB drugs to hypothyroidism in MDR-TB patients, we present a unique case of hypothyroidism in a patient undergoing first-line anti-TB treatment for three months.

## Case presentation

A 17-year-old female patient with a known case of pulmonary tuberculosis (TB) initially presented to emergency with complaints of abdominal pain, distension, and vomiting persisting for 5 days, and a concurrent fever lasting 3 days. Her medical history revealed a positive TB contact history, indicating potential exposure to the infectious agent. The patient was diagnosed with smear positive pulmonary TB based on sputum smear microscopy, which revealed acid-fast bacilli. In addition to her primary symptoms, she reported experiencing constipation, headache, and burning micturition, indicative of systemic involvement. Family history indicated a positive parental history of diabetes mellitus, suggesting a genetic predisposition to metabolic disorders. Prior treatment included a three-month course of intensive anti-tuberculosis therapy (ATT), consisting of daily administration of two tablets before breakfast. Each tablet contained rifampin (150 mg), isoniazid (75 mg), pyrazinamide (400 mg), and ethambutol hydrochloride (275 mg).

Upon examination, the patient’s abdomen was noted to be firm, tender, and distended. Bowel sounds were audible upon auscultation. Chest examination revealed normal vesicular breathing with bilateral clear lung fields. A digital rectal examination (DRE) demonstrated normal anal tone with mild stool staining on the examiner’s finger, suggestive of possible faecal retention. An initial diagnosis of sub-acute intestinal obstruction secondary to pulmonary TB was suspected and subsequently, nasogastric (NG) decompression was performed to alleviate abdominal distension and discomfort. The patient was then started on the following medicines: ceftriaxone (2000 mg), paracetamol (1000 mg), ketorolac tromethamine (30 mg/ml), and omeprazole (40 mg).

Upon laboratory investigations (Table 1), a complex clinical picture was revealed. Haematological analysis demonstrated the presence of anaemia, while serum electrolyte levels were within normal limits. Liver function tests (LFTs) indicated elevated alkaline phosphatase (ALP) levels and decreased albumin levels, suggesting potential liver dysfunction. The malarial parasite immunochromatography technique (MPICT) test returned negative results. Thyroid profile testing initially revealed increased levels of thyroid-stimulating hormone (TSH), with subsequent assessments showing a further rise. However, the lipid profile remained within normal ranges. The initial abdominal ultrasound (US) revealed several significant findings, including a hypoechoic liver, a haemangioma in the right liver lobe, and abnormalities in the left kidney. Additionally, infected ascites and suspected distal small bowel perforation with peritonitis were noted. Following the results of the ascitic tap, the patient was started on meropenem (1000 mg), moxifloxacin (400 mg), metronidazole (500 mg), tramadol (50 mg), and metoclopramide (10 mg). Moreover, histopathological examination of samples from the small and large bowel confirmed the presence of perforation, characterized by dense acute necrotizing inflammation, granulation tissue formation, and extensive fibroblast proliferation. Reactive benign lymph nodes were also identified, with no evidence of malignancy or granuloma detected.

**Table 1:** Various lab parameters on admission.

**Haematology**			
**Component**	**Result**	**Normal Range**	**Units**
Hb	9.0	11.5–15.5	g/dL
MCV	83.2	80.0–100.0	fL
MCH	26.5	27.0–34.0	pg
MCHC	31.8	31.0–36.0	gm/dL
TLC	9.7	4.0–10.0	x10 E 9/L
Lymphocytes	12.0	20.0–40.0	%
Neutrophils	8.0	40.0–80.0	%
Platelets	416	150.0–450.0	x10 E 9/L
CRP	8.9	<0.3	mg/dL
**Coagulation Profile**			
**Component**	**Result**	**Normal Range**	**Units**
PT	16.1	11–13.5	seconds
INR	1.55	<1.1	
**Basic Metabolic Profile**			
**Component**	**Result**	**Normal Range**	**Units**
BUN	2.0	7–20	mg/dL
Cr	0.3	0.7–1.3	mg/dL
Sodium	135	134–144	mEq/L
Potassium	4.8	3.5–5.2	mEq/L
Chloride	101	96–105	mEq/L
Calcium	8.3	8.7–10.2	mg/dL
Magnesium	1.6	1.7–2.2	mg/dL
Phosphate	2.6	2.5–4.5	mg/dL
**Liver Function Tests**			
**Component**	**Result**	**Normal Range**	**Units**
ALT	19	7–55	U/l
ALP	127	40–129	U/l
Total Bilirubin	0.4	0.1–1.2	mg/dL
Albumin	2.6	3.5–5.5	g/dL
**Lipid Profile**			
**Component**	**Result**	**Normal Range**	**Units**
Total Cholesterol	118	Desirable: < 200 Borderline: 200–239 High Risk: 240	mg/dL
Triglycerides	129	Desirable: < 150 Borderline: 150–199 High Risk: 200–499	mg/dL
LDL Cholesterol	51	Desirable: 60–130 Borderline: 130–159 High Risk: 160–189	mg/dL
HDL Cholesterol	41	Desirable: 60 Borderline: 35–45 High Risk: < 35	mg/dL
**Thyroid Profile**			
**Component**	**Result**	**Normal Range**	**Units**
TSH	19.0	0.4–4.5	IU/ml
Free T-3	1.89	2.1–4.4	pg/mL
Free T-4	1.17	0.8–1.8	ng/dL

Based on the initial diagnosis and radiological and histopathological findings, an exploratory laparotomy was performed under general anaesthesia. Operative findings revealed substantial purulent contamination of 2200 ml, cocooning of the omentum, and dense interbowel adhesions. Adhesions were observed between the bowel and the uterus, as well as with the lateral abdominal wall. A serosal tear located 1 foot from the ileocecal junction (ICJ) necessitated exteriorization as a loop ileostomy. A subsequent ultrasound performed within two weeks showed no collection of purulent fluid. Despite being on treatment, her weight continued to decrease compared to her documented weight of 36 kg. The infectious disease department was consulted, and they recommended testing for HIV and repeating the Gene Xpert/MTB-RIF test. The HIV antibody test returned a non-reactive result, indicating the absence of detectable HIV antibodies in the blood sample tested. Additionally, acid-fast bacilli were not visualized on smear microscopy, suggesting the absence of active TB infection. Furthermore, Gene Xpert/MTB-RIF testing yielded negative results for *Mycobacterium tuberculosis* complex, further supporting the absence of tuberculosis infection.

A subsequent operation conducted a week later involved laparotomy and relook under general anaesthesia. Operative findings included the identification and repair of a pinpoint perforation located 2 feet proximal to the stoma site. Multiple interbowel adhesions and jumbled bowel loops were also encountered during the procedure. Additionally, three units of fresh frozen plasma (FFP) were transfused intraoperatively. Interestingly, following the second operation, facial puffiness was observed, prompting a repeat TSH test. The results indicated a significantly higher TSH level compared to the previous measurements. A neck US was recommended, yielding normal results. Consequently, the diagnosis was reassessed and established as hypothyroidism secondary to ATT.

Following the diagnosis of hypothyroidism based on the significantly elevated TSH levels (19 mIU/L), the patient was started on levothyroxine to manage the thyroid dysfunction. The initial dose of levothyroxine was calculated based on the patient’s weight (36 kg) and clinical presentation, following standard guidelines for thyroid hormone replacement in adolescents. A starting dose of 50 mcg/day was selected, which falls within the recommended range of 1.6–1.8 mcg/kg/day (equivalent to approximately 57.6–64.8 mcg/day). This slightly conservative dosing was chosen to avoid overtreatment while ensuring adequate hormone replacement. Thyroid function tests (TFTs) were scheduled for re-evaluation after 6–8 weeks to assess the efficacy of the treatment and guide any necessary dose adjustments. It is important to note that no changes were made to the patient’s ATT regimen. The patient continued to receive the same first-line ATT consisting of rifampin, isoniazid, pyrazinamide, and ethambutol, as hypothyroidism was not deemed a contraindication for continuing these medications. The patient’s thyroid function was expected to stabilise with the initiation of levothyroxine, and ongoing monitoring was planned to ensure effective management of both conditions. Once her symptoms improved, the patient was discharged and instructed to return for follow-up after 8 weeks. Unfortunately, the patient was lost to follow-up, and no further clinical information was available.

## Discussion

The case presentation highlights the complex relationship between TB, its treatment, and subsequent complications, notably hypothyroidism (Table 2). Initially presenting with abdominal pain, distension, vomiting, and fever, the patient’s condition rapidly escalated to severe systemic involvement, culminating in surgical intervention. While pulmonary TB was the primary diagnosis due to smear positivity, the association between pulmonary TB and the intestinal obstruction was established based on clinical suspicion of tuberculosis-related intestinal involvement. Although pulmonary TB rarely manifests with abdominal symptoms, the patient’s intestinal symptoms were considered secondary to potential gastrointestinal TB, given her active pulmonary TB and no other identified causative factors during initial investigations. Therefore, her medical history of pulmonary TB and prior ATT underscore the challenges in managing multi-faceted presentations in TB patients.

**Table 2:** Timeline of events during treatment of the patient.

Date	Event/Investigation	Findings/Notes
10/01/24	Start of ATT (Anti-Tuberculosis Treatment)	Rifampicin 150 mg Isoniazid 75 mg Pyrazinamide 400 mg Ethambutol Hydrochloride 275 mg
15/02/24	Last Menstrual Period (LMP)	
18/02/24	Date of Admission	
19/02/24	Started Medication	Inj. Titan 2 gm BD IV Inj. Provas 1 gm TDS IV Inj. Toradol 30 mg/ml IV Inj. Risek 40 mg OD IV
20/02/24	U/S Whole Abdomen	Hypoechoic liver Haemangioma in right liver lobe Left renal calculus Mild hydronephrosis and hydroureter Infected Ascites Suspected distal small bowel perforation and peritonitis
23/02/24	Ascitic Tap	Raised protein, red blood cells (RBCs), and white blood cells (WBCs) Pus cells (moderate quantity)
24/02/24	Started Medication	Inj. Meropenem 1 g TDS IV Inj. Moxiget 400 mg OD IV Inj. Flagyl 500 mg 8H IV Inj. Tramadol 50 mg TDS IV Inj. Metacolon 10 mg TDS IV
27/02/24	Histopathology	Dense acute necrotizing inflammation with granulation tissue formation Extensive fibroblast proliferation Two reactive benign lymph nodes No evidence of malignancy or granuloma
	Surgery: Exploration and loop ileostomy under general anaesthesia	2200 ml purulent contamination Cocooning of omentum Dense interbowel adhesions Adhesion of bowel with uterus and lateral abdominal wall, 1 ft from ileo-caecal junction (ICJ) serosal tear
28/02/24	Acid-fast bacilli smear and Gene Xpert/MTB-RIF	Acid-fast bacilli not seen; *Mycobacterium tuberculosis* complex (MTB) not seen
02/03/24	U/S Whole Abdomen	No collection seen
04/03/24	Started Medication	Inj. Linezolid 600 mg BD IV Inj. Aminovel BD IV
	HIV Antibody Test	Non-reactive
05/03/24	Surgery: Laparotomy and relook under general anaesthesia	2 ft proximal to stoma site, pinpoint perforation found and repaired Multiple interbowel adhesions and jumbled bowel loops
06/03/24	Noted facial puffiness, thyroid stimulating hormone (TSH) test	Significantly high TSH
11/03/24	US Neck	Normal
12/03/24	Levothyroxine initiated	

Multidrug-resistant tuberculosis (MDR-TB) is characterized by its resistance to isoniazid and rifampin, the two most effective drugs used to treat TB [5]. While hypothyroidism is traditionally considered a rare side effect of TB treatment, emerging evidence suggests its prevalence is increasing among MDR-TB patients. A meta-analysis involving 30 studies and over 6000 patients reported a 17.0% prevalence of hypothyroidism among those receiving MDR-TB treatment [6]. Local data from Baluchistan further corroborates this trend, indicating a hypothyroidism prevalence of 21.5% in MDR-TB patients [7].

The patient initially presented with symptoms including abdominal pain, distension, vomiting, and fever, which were primarily attributed to pulmonary TB and its associated complications. The initial working diagnosis was subacute intestinal obstruction potentially secondary to gastrointestinal TB. This was supported by the presence of infected ascites and the findings of small bowel perforation and peritonitis during laparotomy. Interestingly, there was no evidence of granulomas or acid-fast bacilli on histopathological examination, which limited confirmation of gastrointestinal TB. A complication of ATT was not suspected at this stage as the patient was still in the early phase of treatment, and no literature strongly associates first-line ATT with intestinal obstruction or perforation. Moreover, these symptoms were not immediately indicative of hypothyroidism. However, following initial management and surgical intervention, the patient developed facial puffiness, prompting a repeat TSH test. The subsequent marked increase in TSH levels, in conjunction with a normal neck ultrasound, led to the diagnosis of hypothyroidism. In considering the aetiology, other potential causes of hypothyroidism were systematically excluded. The patient had no family history of thyroid disease, nor did she present with any autoimmune conditions such as type 1 diabetes or caeliac disease. Additionally, there was no history of prior treatment for hyperthyroidism, exposure to neck or upper chest radiation, or thyroid surgery. These exclusions significantly strengthen the hypothesis that the hypothyroidism observed in this case was an adverse effect of first-line ATT, which the patient had been receiving for three months at the time of diagnosis. Although the patient was diagnosed with hypothyroidism based on elevated TSH levels, the absence of classic hypothyroidism symptoms such as weight gain, fatigue, or cold intolerance raises the possibility of sick euthyroid syndrome (SES). SES is commonly observed in critically ill patients and is characterized by abnormal thyroid function tests without intrinsic thyroid dysfunction. The patient’s persistent weight loss and severe systemic illness make SES a potential alternative diagnosis that warrants consideration. However, the slightly reduced free T3 level and a normal free T4 level, along with elevated TSH supports the diagnosis of primary hypothyroidism rather than SES. The patient’s clinical picture underscores the critical need for vigilant monitoring of thyroid function in individuals undergoing anti-TB therapy. The correlation between hypothyroidism and ATT, particularly in MDR-TB cases, necessitates a proactive approach to thyroid monitoring. Chhabra et al. conducted a study to assess thyroid function in patients undergoing treatment for MDR-TB. The study specifically focused on the impact of second-line anti-tuberculosis drugs, such as para-aminosalicylic acid (PAS) and ethionamide (Eto), on TFTs. According to the results, both PAS and Eto were identified as the major contributors to the development of hypothyroidism among MDR-TB patients [8]. Since the study focused on second-line treatments and their thyroid-related side effects, it demonstrated that hypothyroidism is more commonly associated with these drugs rather than with first-line therapies. However, it is important to note that the absence of detailed analysis on first-line drugs does not necessarily rule out the possibility of thyroid dysfunction as a side effect; rather, it highlights the greater risk associated with second-line therapies. Similarly, another study revealed the relationship between hypothyroidism and second-line anti-TB agents [3]. In our case report, the patient was never on second-line drugs.

Based on existing literature [1], hypothyroidism associated with ATT—particularly second-line medications—is often reported to develop after two to three months of treatment, with resolution occurring four to six weeks after cessation. Therefore, the timeline of hypothyroidism emerging at three months (refer to Table 2) fits well with previous reports, especially in the context of MDR-TB treatment. However, because our case involves first-line ATT, it is important to highlight that while hypothyroidism linked to second-line drugs is well-documented, fewer cases detail this side effect for first-line agents and the development of hypothyroidism three months into first-line ATT in our patient may represent an under-recognised adverse effect. Moreover, our case report details the diagnostic process for hypothyroidism, primarily relying on elevated TSH levels and normal neck ultrasound findings. These are the standard diagnostic tools for hypothyroidism, regardless of aetiology [9]. Studies like those by Quiroz-Aldave et al. [2] and Chhabra et al. [8] employed TSH and free T4 testing to confirm hypothyroidism in MDR-TB patients. These methods are consistent with our approach. It is, therefore, recommended that thyroid hormone levels be assessed before starting anti-TB treatment and subsequently every three to six months. This protocol ensures timely intervention with levothyroxine to mitigate the risk of subclinical or overt hypothyroidism. Early detection and treatment can prevent the exacerbation of symptoms and improve overall patient outcomes [3]. While diagnostic practices are consistent with previous studies on second-line ATT, the rarity of first-line-associated hypothyroidism necessitates further research to establish more robust guidelines.

The unique intricacy of this case, followed by extensive surgical interventions and chronic systemic issues, underscores the significance of adopting a multidisciplinary approach. The active involvement of infectious disease specialists, endocrinologists, and surgeons was crucial in addressing the various dimensions of the patient’s condition. The patient’s negative test results for HIV and *M. tuberculosis* complex emphasise the value of comprehensive diagnostic assessments to eliminate other possible causes of her symptoms and direct the implementation of suitable treatment plans.

To emphasise the necessity of regular thyroid function monitoring in patients with TB, particularly those with MDR-TB, this case underscores the importance of proactive measures. Given the increasing prevalence of hypothyroidism in this population, early detection and timely management with levothyroxine are paramount. Not only does this improve patients’ quality of life, but it also ensures the effectiveness of TB treatment regimens by preventing additional complications.

## Conclusion

We present the case of a 17-year-old female patient which highlights the critical need for vigilant monitoring of thyroid function in patients undergoing anti-TB therapy, including first-line treatments. Despite extensive treatment and surgical interventions for severe systemic complications of TB, the patient developed hypothyroidism, suggesting the monitoring of hypothyroidism among TB patients. Regular thyroid function assessments and timely management with levothyroxine can prevent subclinical and overt hypothyroidism, improve patient outcomes, and ensure the effectiveness of TB treatment regimens. A multidisciplinary approach is essential in managing the complex presentations and complications associated with TB.
